# Prediction of complications in early-onset pre-eclampsia (PREP): development and external multinational validation of prognostic models

**DOI:** 10.1186/s12916-017-0827-3

**Published:** 2017-03-30

**Authors:** Shakila Thangaratinam, John Allotey, Nadine Marlin, Julie Dodds, Fiona Cheong-See, Peter von Dadelszen, Wessel Ganzevoort, Joost Akkermans, Sally Kerry, Ben W. Mol, Karl G. M. Moons, Richard D. Riley, Khalid S. Khan, for the PREP Collaborative Network, Raajkumar Sundararajah, Avideah Nejad, Rehan Khan, Celia Burrell, Manish Gupta, Vincent Oon, Rezan Kadir, Zeudi Ramsey-Marcelle, Louise Page, Baskaran Thilaganathan, Bill Martin, Shagaf Haj Bakour, Hassan Morsi, David Churchill, Fidelma O’Mahony, Karen Powell, Jayasree Srinivasan, Michele Mohajer, Siobhan Quenby, Lakshmi Thirumalaikumar, Justin Konje, Jim Thornton, George Bugg, Shonag Mackenzie, Aarti Ullal, Marie Smith, Rita Arya, Simon Cunnigham, James Walker, Nigel Simpson, Joanne Page, Claire Oxby, Karen Watkins, Derek Tuffnell, S. Bober, A. Wijesiriwardana, Helene Brandon, Saif El-Badawy, Sara Brigham, Lanre Shorinola, Aethele Khunda, Shaku Kalla, Mohammed M. Abdullah Agha, Stephen Poku, Ayo Olawo, Johnson Amu, Philip Banfield, Franz Majoko, Julia Alcide, Jyothi Rajeswary, Marwan Salloum, Alexandra Rees, Odiri Oteri, Sunday Ikhena, Janet Creswell, Feroza Dawood, Umber Agarwal

**Affiliations:** 1 Women’s Health Research Unit, Barts and the London School of Medicine and Dentistry, Queen Mary University of London, London, UK; 2 Multidisciplinary Evidence Synthesis Hub (mEsh), Queen Mary University of London, London, UK; 3 Pragmatic Clinical Trials Unit, Blizard Institute, Barts and the London School of Medicine and Dentistry, Queen Mary University London, London, UK; 4 Institute of Cardiovascular and Cell Sciences, St George’s, University of London, London, UK; 5 Departments of Obstetrics and Gynecology, University of Amsterdam, Academic Medical Center, Amsterdam, The Netherlands; 6 Department of Obstetrics, Leiden University Medical Center, Leiden, The Netherlands; 7 The Robinson Research Institute, School of Paediatrics and Reproductive Health, University of Adelaide, Adelaide, Australia; 8 The South Australian Health and Medical Research Institute, Adelaide, Australia; 9 Julius Centre for Health Sciences and Primary Care, University Medical Centre Utrecht, Utrecht, The Netherlands; 10 Research Institute for Primary Care and Health Sciences, Keele University, Keele, Staffordshire UK

**Keywords:** Early-onset, Pre-eclampsia, Prognostic models, Maternal, Complications

## Abstract

**Background:**

Unexpected clinical deterioration before 34 weeks gestation is an undesired course in early-onset pre-eclampsia. To safely prolong preterm gestation, accurate and timely prediction of complications is required.

**Method:**

Women with confirmed early onset pre-eclampsia were recruited from 53 maternity units in the UK to a large prospective cohort study (PREP-946) for development of prognostic models for the overall risk of experiencing a complication using logistic regression (PREP-L), and for predicting the time to adverse maternal outcome using a survival model (PREP-S). External validation of the models were carried out in a multinational cohort (PIERS-634) and another cohort from the Netherlands (PETRA-216). Main outcome measures were C-statistics to summarise discrimination of the models and calibration plots and calibration slopes.

**Results:**

A total of 169 mothers (18%) in the PREP dataset had adverse outcomes by 48 hours, and 633 (67%) by discharge. The C-statistics of the models for predicting complications by 48 hours and by discharge were 0.84 (95% CI, 0.81–0.87; PREP-S) and 0.82 (0.80–0.84; PREP-L), respectively. The PREP-S model included maternal age, gestation, medical history, systolic blood pressure, deep tendon reflexes, urine protein creatinine ratio, platelets, serum alanine amino transaminase, urea, creatinine, oxygen saturation and treatment with antihypertensives or magnesium sulfate. The PREP-L model included the above except deep tendon reflexes, serum alanine amino transaminase and creatinine. On validation in the external PIERS dataset, the reduced PREP-S model showed reasonable calibration (slope 0.80) and discrimination (C-statistic 0.75) for predicting adverse outcome by 48 hours. Reduced PREP-L model showed excellent calibration (slope: 0.93 PIERS, 0.90 PETRA) and discrimination (0.81 PIERS, 0.75 PETRA) for predicting risk by discharge in the two external datasets.

**Conclusions:**

PREP models can be used to obtain predictions of adverse maternal outcome risk, including early preterm delivery, by 48 hours (PREP-S) and by discharge (PREP-L), in women with early onset pre-eclampsia in the context of current care. They have a potential role in triaging high-risk mothers who may need transfer to tertiary units for intensive maternal and neonatal care.

**Trial registration:**

ISRCTN40384046, retrospectively registered.

**Electronic supplementary material:**

The online version of this article (doi:10.1186/s12916-017-0827-3) contains supplementary material, which is available to authorized users.

## Background

Early-onset pre-eclampsia presents with hypertension and proteinuria before 34 weeks’ gestation, and contributes disproportionately to pregnancy complications compared to late-onset disease [1–4]. Complications necessitate intensive care in a third of women, who are at risk of unexpected clinical deterioration [5, 6]. The only known cure for the condition is delivery of the baby [7], which often occurs at extreme preterm gestation, and requires immediate neonatal intensive care.

A diagnosis of early onset pre-eclampsia with impending complications, including iatrogenic preterm delivery before 34 weeks, often triggers transfer to a tertiary unit with intensive care facilities for the mother and the preterm infant. Given the paucity of neonatal intensive care beds and high-dependency space for mothers in tertiary centres [8] and the prolonged time and significant resources required to facilitate the process [9, 10], an accurate estimation of risk at various time points after diagnosis of early onset pre-eclampsia is needed to prioritise and plan care. Information on the mother’s overall risk status will determine the intensity of monitoring in pregnancy.

Existing models on pre-eclampsia have not been designed specifically to predict complications in early-onset disease and do not have a sufficient sample size to robustly predict risks at various time points [1–4] due to the small numbers of events per variable [3, 4, 11]. The adverse outcomes predicted by the models do not include risk of preterm delivery, an important component that influences decisions regarding in utero transfer [4]. Furthermore, the models do not take into account the effect of treatment (including early delivery), which may influence the choice of predictors and performance of models [12].

We developed multivariable prognostic models for providing individual risks of adverse maternal outcomes, including delivery of preterm infant before 34 weeks, in women with early-onset pre-eclampsia in the UK, by 48 hours and by discharge. We externally validated these in various independent datasets across the world [4, 11].

## Methods

We conducted the study using a prospective protocol [13] and reported in line with the TRIPOD (Transparent Reporting of a multivariable prediction model for Individual Prognosis Or Diagnosis) recommendations [14–16]. A panel of experts provided additional methodological input on the development of prediction models [17]. The National Research Ethics Service Committee (West Midlands, UK) provided approval (11/WM/0248.)

### Population

We approached potentially eligible mothers from 53 obstetric units within secondary and tertiary care hospitals in the UK. Information about the study was provided when women attended the hospital for obstetric care. We recruited consecutive women with suspected or confirmed pre-eclampsia before 34 weeks’ gestation from December 2011 to April 2014. Women with confirmed early-onset pre-eclampsia contributed to model development (Additional file 1: Table S1). Women were excluded from the study if the outcome of interest had occurred prior to the assessment of predictors, there was insufficient time to obtain informed consent or there was a lack of translators for non-English speaking mothers. All women in the study were managed according to National Institute for Health and Care Excellence guidelines on Hypertension in Pregnancy [18].

### Predictors

An initial list of 33 candidate predictors was identified from published systematic reviews [19–24] and primary studies [4, 25–30], and subsequently (before any data analysis) reduced to 22 after prioritising their importance by a Delphi consensus among experts [22]. Candidate predictors included maternal characteristics, relevant medical history, symptoms, signs, investigations and interventions that have the potential to modify the probability of outcome (Table 1). In cases of multiple collected values for the same predictor, we chose the worst value within the first 24 hours. We also considered predictors for fetal outcomes shown in Additional file 1: Table S2.

**Table 1:** Details of candidate predictors of women in the PREP study and the proportion with missing values

Candidate predictor	Women with early onset pre-eclampsia *n* = 954 Mean (SD) or *n* (%)	Number of women with missing data *n* (%)
Maternal characteristics		
Maternal age (years), mean (SD)	30.2 (6.1)	2 (0.2%)
Gestational age at diagnosis (weeks), mean (SD)	30.5 (2.9)	–
Number of fetuses in pregnancy^a^		
Singleton	866 (91%)	
Twins	83 (9%)	–
Triplets	5 (1%)	
Parity		–
0	551 (58%)	
1	207 (22%)	
2	109 (11%)	
3	55 (6%)	
4	20 (2%)	
5 – 9	12 (1%)	
History		
Medical history^b^		1 (0.1%)
None	601 (63%)	
At least one condition	251 (26%)	
Two or more conditions	101 (11%)	
Chronic hypertension	139 (15%)	10 (1.0%)
Renal disease	30 (3%)	10 (1.0%)
Previous history of pre-eclampsia	169 (43%)	558^b^
Autoimmune disease	18 (2%)	32 (3.4%)
Diabetes mellitus	109 (11%)	6 (0.6%)
Symptoms		
Symptoms of headache and/or visual disturbance	382 (41%)	28 (2.9%)
Symptoms of epigastric pain, nausea and/or vomiting	202 (22%)	47 (4.9%)
Symptoms of chest pain and/or breathlessness	60 (7%)	126 (13.2%)
Bedside examination and tests		
Systolic blood pressure (mmHg), mean (SD)	159 (19)	5 (0.5%)
Diastolic blood pressure (mmHg), mean (SD)	99 (12)	5 (0.5%)
Clonus	95 (17%)	403 (42.2%)
Exaggerated tendon reflexes	147 (24%)	353 (37%)
Oxygen saturation by pulse oximetry (%), mean (SD)	98 (2)	521 (54.6%)
Oxygen saturation: abnormal (< 94%)	4 (1%)	521 (54.6%)
Urine dipstick		
None/Trace	39 (4%)	
1+	170 (18%)	
2+	314 (34%)	19 (2%)
3+	306 (33%)	
≥ 4	106 (11%)	
Laboratory tests		
Haemoglobin (g/L), mean (SD)	11.9 (1.3)	37 (3.9%)
Platelet count (× 10^9^/L), mean (SD)	226 (78)	41 (4.3%)
Alanine transaminase (U/L), mean (SD)	31.0 (71.0)	75 (7.9%)
Serum uric acid (μmol/L), mean (SD)	0.6 (2.7)	165 (17.3%)
Serum urea (mmol/L), mean (SD)	4.6 (4.4)	70 (7.3%)
Serum creatinine (μmol/L), mean (SD)	61.0 (17.8)	38 (4%)
Urine PCR (mg/mmol), mean (SD)	273 (492)	109 (11.4%)
Treatment provided		
Any anti-hypertensive therapy^c^	753 (79%)	6 (0.6%)
Oral anti-hypertensive therapy	734 (77%)	6 (0.6%)
Parenteral anti-hypertensive therapy	111 (12%)	6 (0.6%)
Parenteral magnesium sulfate^d^	144 (15%)	6 (0.6%)

^a^Number of pregnancies

^b^All missing values are for nulliparous women where previous occurrence of pre-eclampsia is not applicable

^c^On-going at diagnosis or introduced within 1 day of diagnosis

^d^Administered any time before diagnosis or within 24 h of diagnosis

### Outcomes

An independent panel of experts ranked the outcomes for their importance to clinical practice [17]. Based on the PIERS study, the components of the outcome were identified by a Delphic consensus [3]. We defined the primary outcome as maternal complication that included maternal death, neurological, hepatic, cardiorespiratory, renal or haematological complications, or delivery before 34 weeks (Additional file 1: Table S3a). The panel agreed that delivery before 34 weeks is often offered for medical reasons to avoid complications as per the national guidelines; thus, excluding this as an adverse outcome would underestimate the true incidence of adverse outcomes and lead to prognostic models that yield too low risk predictions of actually developing an adverse outcome [17]. Hence, deliveries before 34 weeks’ gestation were added as a component of the primary outcome before data analysis. The secondary outcomes included composite perinatal outcomes by discharge, which were also developed by Delphi consensus (Additional file 1: Table S3b) [3].

### Sample size

Based on systematic reviews on accuracy of tests in predicting complications in women with pre-eclampsia [3, 19, 23, 24], we expected 20% of women with early-onset pre-eclampsia to develop adverse maternal outcome at any time point, and about 10 events were required for each candidate predictor to reduce model overfitting issues [31]. We revised our initial sample size of 500 women, to continue recruitment until 100 adverse maternal events were reached. Prior to analysis, discussions with the independent Steering Committee resulted in extension of the primary outcome to also include delivery before 34 weeks’ gestation [17] (Additional file 1: Table S4).

### Datasets for external validation

We externally validated the developed PREP models in two independent datasets, namely the PIERS (Pre-eclampsia Integrated Estimate of RiSk) study [4], a prospective cohort on prediction of adverse maternal outcomes in women with any-onset pre-eclampsia (634 women recruited in Canada, New Zealand, Australia and the UK), and the PETRA (Pre-Eclampsia TRial Amsterdam) study, a randomised trial on effectiveness of plasma expansion in the management of early-onset hypertensive disease in pregnancy (216 women) [11].

### Data analysis

#### Model development

We developed two prediction models – a survival model (PREP-S) censored at 34 completed weeks’ gestation to predict the risk over time at daily intervals from diagnosis of early onset pre-eclampsia, and a logistic regression model (PREP-L) to predict the overall risk of maternal complications by postnatal discharge. We evaluated the performance of the models in terms of calibration and discrimination. Calibration assesses if the predicted risks agree with the observed risks. We reported this graphically using calibration plots and estimated the calibration slope (with 95% confidence intervals) across the spectrum of predicted risks for included women; a calibration slope of 1 is desired. In addition to calibration slopes for both models, we calculated the ratio of observed to predicted probability of outcome for the PREP-S model in various risk groups at 48 hours, 1 week and overall. Discrimination indicates how well the model separates mothers without complications from those with complications, and was quantified using Harrell’s C-statistic for the PREP-S model, and the generic C-statistic for the PREP-L model with 95% confidence intervals (CI). A C-statistic of 0.5 indicates no discrimination beyond chance, whereas a C-statistic of 1 indicates perfect discrimination.

We used the Royston–Parmar approach for the survival analysis (PREP-S) [32–34] to model the cumulative baseline hazard scale using restricted cubic splines [35]. The numbers of knots in the spline function were chosen based on visual inspection and goodness-of-fit statistics [32–34].

During development of both models, we used the ICE package in Stata with five imputations to deal with missing predictor values under a missing at random assumption [35]. The estimates across imputed datasets were combined using Rubin’s rules to produce final parameter estimates for the model [36]. No outcomes were imputed. Sensitivity analysis examined the impact of increasing the number of imputed datasets to 40.

For each PREP-S and PREP-L models separately, we started with the full model (including all candidate predictors) and used backwards selection to identify predictors to include at *P* < 0.15 of the log likelihood ratio test. We forced gestational age and maternal age at diagnosis into the models to ensure clinical acceptability of the final model. We evaluated non-linear trends of the continuous predictors using multivariable fractional polynomials, with *P* < 0.01 (for the change in model fit) used to justify the non-linear trends. We reported the model performance at 48 hours, 1 week and overall. A sensitivity analysis was conducted by adding oral and parenteral anti-hypertensives separately into the final models to check if model fit was improved by using a likelihood ratio test with *P* < 0.15. We checked the final PREP-S model for time dependent effects (non-proportional hazards) of included predictors.

#### Internal validation and adjustment for over-optimism

We internally validated the performance of our models. We estimated their apparent (i.e. before adjustment for model overfitting) calibration and discrimination, by averaging across performance across imputed datasets. We used non-parametric bootstrapping to estimate over-optimism in performance, where in each of 100 bootstrap samples we repeated the entire modelling process (including predictor selection) and compared apparent model performance (in bootstrap sample) with performance in the original sample. The average optimism across all bootstrap samples was then used to calculate, for our developed models, their optimism-adjusted C-statistics and optimism-adjusted calibration slopes. Based on the latter, uniform shrinkage factors were applied to all the predictor effects in the final developed models to account for the over-optimism identified. The choice of imputed dataset to obtain bootstrap samples from made little difference to the optimism estimates.

#### External validation

For assessment of model transportability, the final optimism-adjusted models were externally validated in the PIERS and PETRA datasets. However, as some predictors in the final models were not available in the external datasets, we developed a reduced version of our final models. That is, we re-estimated the available predictor effects and intercept terms within the models using the development data, and adjusted for optimism according to the method described above. These reduced PREP models were then validated in the PIERS and PETRA population.

To examine calibration graphically, women were grouped into four risk categories, namely low (<15th centile), intermediate (15–50th), high (> 50–85th) and very high (> 85th) in the PREP-S model, and into tenths of predicted risk (defined by deciles) in the PREP-L model. If necessary, we examined whether recalibration of the intercepts of the developed models improved their performance in the validation sets.

### Prediction of secondary (adverse perinatal) outcomes

A logistic regression model was used to predict the risk of adverse perinatal outcomes, as for the maternal outcomes. In addition to the maternal predictors, we evaluated cardiotocography and uterine artery Doppler. Pregnancy was the unit of analysis and non-linear terms were not considered. A similar variable selection process on the full list of maternal and fetal candidate predictors was done.

All analyses were carried out using Stata version 12.0 [35].

## Results

**Fig. 1:**
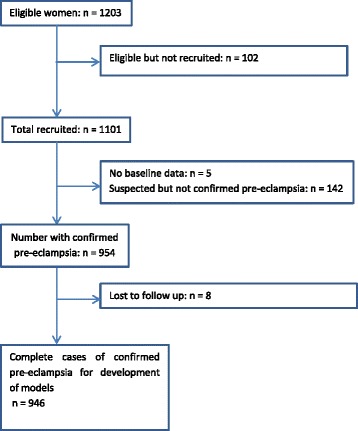
Flow of women recruited in the PREP study for development of prediction model(s) for adverse maternal outcomes

We screened 3302 pregnant women, of whom 1101 were recruited to the study. There were 954 women with confirmed early-onset pre-eclampsia. We included 946 women with complete outcome data in the final prediction models (Fig. 1).

### Characteristics of women in the PREP study

Over 90% (866/954) of women with early-onset pre-eclampsia had new onset disease. More than half of all women were nulliparous (551/954, 58%) and the mean gestational age of diagnosis was 30.5 (SD 2.9) weeks. More than three-quarters of women were on anti-hypertensives at baseline (753/948, 79%). Of the 15% of mothers who received magnesium sulfate at baseline in the PREP study, the rationale was documented as severe pre-eclampsia in 72% (104/144), and not reported in the rest (Table 1).

### Outcomes

About a fifth of mothers diagnosed with the condition suffered a complication (169/946, 18%) by 48 hours after diagnosis of early onset pre-eclampsia, and two-thirds (633/946, 66.9%) by postnatal discharge. The most frequent outcome was early preterm delivery before 34 weeks gestation (580/946, 61.3%), followed by postpartum haemorrhage (74/946, 7.8%) (Additional file 1: Table S5a). Three-quarters of all neonates (702/945, 74%) had suffered at least one complication by discharge, with admission to the neonatal intensive care unit being the most common outcome in 72% (681/945) (Additional file 1: Table S5b).

### Model development

#### PREP-S model: risks at various time points

The apparent and optimism-adjusted Harrell’s C-statistic of the developed PREP-S model were 0.77 (95% CI, 0.75–0.79) and 0.75 (95% CI, 0.73–0.78), respectively. The optimism-adjusted C-statistic of the model by 48 hours and 1 week was 0.84 (95% CI, 0.81–0.87) and 0.79 (95% CI, 0.76–0.81), respectively. The final predictors included in the PREP-S model are listed in Box 1.

**Table:** 

**Box 1: Full PREP prognostic models to calculate the risk of adverse maternal outcomes in women with early onset pre-eclampsia** **a. Risk at various time points from diagnosis until 34 weeks’ gestation using the survival model (PREP-S)** S_(t)_ = S_0_ (t)^§^ ^exp ((β_1_*X_1_ + ⋯ + β_n_*X_n_)) S_(t)_ = S_0_(t)^exp(– 0.031*maternal age + 1.514*((Log(GA at diagnosis/10))^–2^ – 0.8345136) + 5.707*((Log(GA at diagnosis/10))^–2^* ln(log(GA at diagnosis/10)) – 0.0652155) + 0.122 (exaggerated tendon reflexes) – 0.169 (one pre-existing medical condition) – 0.384 (two or more pre-existing medical conditions) + 0.016*systolic blood pressure + 0.797 (oxygen saturation < 94% on air) – 0.002*platelet count + 0.126*log(alanine amino transferase) + 0.605*log(serum urea)^2^ – 0.144*log(serum urea)^3^ + 0.265*log(serum creatinine) + 0.080*log(protein creatinine ratio) + 0.176 (baseline treatment with any antihypertensive) + 1.066 (baseline treatment with magnesium sulfate)) § *S*_*0*_ *(t) – baseline survival adjusted for optimism at time t S*_*0*_ *(48 hrs) = 0.99142, S*_*0*_ *(72 hrs) = 0.98542, S*_*0*_ *(1 week) = 0.96492, S*_*0*_ *(1 month) = 0.87377* **b. Overall risk by postnatal discharge using the logistic model (PREP-L)** Probability (maternal adverse outcome) = exp(X)/(1 + exp(X)), Where X = – 1.507– 0.020*maternal age + 12.052*(log (gestational age))^3^ – 39.90241) – 7.930*((log (gestational age))^3^*log(log (gestational age) – 49.08188) – 0.330 (if one pre-existing medical condition) – 0.579 (if two or more pre-existing medical conditions) + 0.146*log (urine protein creatinine ratio) – 0.951*(log (serum urea)^–1^) – 0.004*platelet count + 0.024*systolic blood pressure + 0.409 (baseline treatment with antihypertensive) + 1.252 (baseline treatment with magnesium sulfate) *Predictor value is 1 when present and 0 when absent*	

**Fig. 2:**
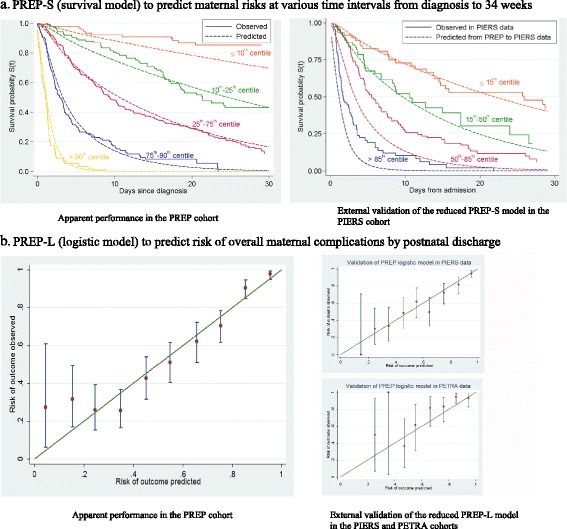
Apparent performance and calibration in the external cohorts of the PREP models for predicting adverse maternal outcomes in early onset pre-eclampsia

Figure 2 shows the model-based mean survival curves for these prognostic groups compared to their observed Kaplan–Meier survival curves up to 1 month after diagnosis. Overall, there was excellent apparent agreement, both at 48 hours and by 1 week after delivery, across all risk categories. In the highest risk group (> 90th centile), 81% of mothers experienced the adverse outcome by 48 hours after diagnosis and 96% by 1 week. In the lowest risk group (≤ 10th centile), adverse maternal outcome rate was 2% by 48 hours after diagnosis and 7% by 1 week. The bootstrap approach showed an optimism of 0.14 in calibration slope, and thus we reduced the predictor effect estimates within the final model by the uniform shrinkage factor of 0.86 (Box 1). Sensitivity analysis (e.g. increasing the number of imputations for missing data from 5 to 40) identified no important changes in the developed model or its C-statistic.

#### PREP-L model: risk by postnatal discharge

The apparent C-statistic for the PREP-L model was 0.84 (95% CI, 0.82–0.87) and following bootstrap adjustment for optimism it was 0.82 (95% CI, 0.80–0.84) (Table 2). The final predictors included in the logistic PREP-L model are listed in Box 1. The apparent calibration plot in Fig. 2 shows mostly good agreement between predicted versus observed risks. We identified an optimism of 0.14 for the calibration slope, and thus reduced the predictor effect estimates within the final developed model by the uniform shrinkage factor of 0.86 (Box 1). Sensitivity analyses did not identify any important changes.

**Table 2:** Performance of the PREP-S (risk at various time points) and PREP-L (overall risk by discharge) models in the derivation cohort and the external validation datasets for prediction of complications in women with early onset pre-eclampsia

Model performance	Development cohort	External cohorts	
	PREP	PIERS	PETRA
PREP-S model	N = 946	N = 339	
C-statistic (95% CI)			–
At 48 hours	0.84 (0.81–0.87)^b^	0.75 (0.69–0.81)^a^	
At 1 week	0.79 (0.76–0.81)^b^	0.72 (0.68–0.76)^a^	
Overall	0.75 (0.73–0.78)^b,c^	0.71 (0.67–0.75)^a^	
Calibration slope (95% CI)			–
At 48 hours	1	0.80 (0.62–0.99)	
At 1 week	1	0.75 (0.61–0.89)	
Overall	1	0.67 (0.56–0.79)	
PREP-L model	N = 946	N = 437	N = 211
C-statistic (95% CI)	0.82 (0.80, 0.84)^b,d^	0.81 (0.77–0.85)^a^	0.75 (0.64–0.86)^a^
Calibration slope (95% CI)	1	0.93 (0.72–1.13)	0.90 (0.48–1.32)

*CI* confidence interval; *N* number of women analysed; *PREP* PRediction of complications in Early-onset Pre-eclampsia study; *PIERS* Pre-eclampsia Integrated Estimate of RiSk study; *PETRA* Pre-Eclampsia TRial Amsterdam study

^a^Apparent C-statistic

^b^Optimism adjusted C-statistic

^c^Overall apparent C-statistic 0.77 (95% CI 0.75, 0.79)

^d^Apparent C-statistic 0.84 (95% CI, 0.82–0.87)

### External validation of PREP models

In the external datasets, data were available for 636 (PIERS study) and 216 women (PETRA study) with early-onset pre-eclampsia (Additional file 1: Table S6). The reduced version of the PREP-S (without serum urea and deep tendon reflex) and PREP-L (without serum urea) models were evaluated in the PIERS and PETRA cohorts.

#### PREP-S model performance in external datasets

##### Calibration

The calibration slopes at 48 hours, 1 week and overall were 0.80 (95% CI, 0.62–0.99), 0.75 (95% CI, 0.61–0.89) and 0.67 (95% CI, 0.56–0.79), respectively, on validation in the PIERS external dataset (Table 2). There was good calibration between observed and predicted probability of survival without complications in the low- (91% vs. 95%) and intermediate-risk (88% vs. 89%) groups at 48 hours, but with under prediction of survival in the high- (90% vs. 70%) and very high-risk (46% vs. 28%) groups (Table 3).

**Table 3:** Comparison of observed and predicted probability of survival using PREP-S model at 48 hours and by 1 week after diagnosis of early onset pre-eclampsia in external datasets

Risk stratification (No. of women)	Time	External validation in PIERS cohort		
		Observed survival probability $\left(\overline{S}(t)\right)$	Predicted survival probability (*Ŝ*(*t*))	Ratio $\left(\overline{S}(t)\right)/\left(\widehat{S}(t)\right)$
≤ 15th (n = 59)	48 hours	0.91	0.95	0.96
	1 week	0.81	0.79	1.0
> 15th–50th (n = 70)	48 hours	0.88	0.89	1.0
	1 week	0.62	0.60	1.0
> 50th –85th (n = 123)	48 hours	0.90	0.70	1.3
	1 week	0.40	0.23	1.7
> 85th (n = 87)	48 hours	0.46	0.28	1.6
	1 week	0.14	0.02	7.0

##### Discrimination

The C-statistic of the reduced PREP-S model was 0.75 (95% CI, 0.69–0.81) at 48 hours, 0.72 (95% CI, 0.68–0.76) at 1 week after diagnosis and 0.71 (95% CI, 0.67–0.75) overall. The performance of the PREP-S model could not be evaluated in the PETRA dataset due to non-availability of data on time to event.

#### PREP-L model performance in external datasets

The reduced PREP-L model showed excellent agreement between predicted and observed risks, with a calibration slope of 0.93 (95% CI, 0.72–1.13). The C-statistic of 0.81 (95% CI, 0.77–0.85) in the PIERS dataset (Table 2 and Fig. 2) was similar to that observed in the development data, and the reduced model separates women into low-, intermediate-, high- and very high-risk groups in the PIERS cohort similar to the PREP data. Recalibration of the intercept term to the PIERS data did not improve the calibration slope. In the PETRA dataset, the reduced model had good calibration (0.90; 95% CI, 0.48–1.32), though with slight over-prediction in most tenths (Fig. 2), and a slightly lower C-statistic than before (0.75; 95% CI, 0.64–0.86).

### Risk of adverse perinatal outcomes

An increase in the gestational age at diagnosis of pre-eclampsia and any pre-existing medical history were associated with a reduction in the odds of perinatal complications (OR, 0.91; 95% CI, 0.39–0.99 and OR, 0.65; 95% CI, 0.44–0.98, respectively). Raised urine protein:creatinine ratio (OR, 1.29; 95% CI, 1.11–1.50), serum urea (OR, 1.72; 95% CI, 1.07–2.76), management with anti-hypertensives (OR, 1.56; 95% CI, 1.04–2.37), treatment with magnesium sulfate (OR, 2.40; 95% CI, 1.04–5.57), abnormal uterine artery Doppler (OR, 1.94; 95% CI, 1.08–3.51), and when the expected fetal weight was less than 10th centile by ultrasound (OR, 2.54; 95% CI, 1.46–4.40) were all associated with an increase in the odds of complications (Additional file 1: Table S2).

## Discussion

We have developed and validated models to predict maternal complications, including preterm delivery, in women with early onset pre-eclampsia. The models are based on routine tests, which are performed in clinical practice on suspicion or confirmation of pre-eclampsia. The PREP-S model can be used at the point of care to predict the risks of complications at various time points, including 48 hours, whereas the PREP-L can be used to provide overall risk by postnatal discharge. The apparent performances of the models show good discrimination at 48 hours, and by discharge. In terms of discrimination performance in external datasets, the C-statistic estimates for reduced models were similar to those observed in the PREP data for all models. For calibration performance in external datasets, the reduced PREP-L and PREP-S models had some over-prediction with calibration slopes less than 1, but miscalibration was only slight for PREP-L by discharge and PREP-S by 48 hours.

### Strengths and limitations

We developed the PREP models by using a large, well-defined and prospectively collected cohort of mothers from several centres in the UK. We clearly defined the predictors, and only used values within 24 hours of diagnosis of early onset pre-eclampsia, to allow application of the model at the point of diagnosis. The final models included clinically relevant predictors used in practice to assess disease severity, such as blood pressure, thereby providing face validity. Missing values of predictors were dealt with multiple imputation to avoid loss of useful information. Overfitting issues were reduced by a large sample size and through optimism-adjustment methods.

We identified the components of the composite outcome by a Delphi survey of experts, and prioritised for their clinical importance, thereby ensuring that only the clinically most relevant outcomes were included. An a priori expert workshop provided guidance to minimise bias from treatment paradox. We addressed this problem by including administration of anti-hypertensives and magnesium sulfate as predictors, and delivery before 34 weeks as an outcome [17]. In women with severe pre-eclampsia, magnesium sulfate is administered either for prevention of eclamptic seizures, or more recently, for neuroprotection of the preterm infant [37]. However, we expect the proportion of women who received it solely for fetal neuroprotection to be small, and less likely to significantly alter the performance of the models. We had sufficient sample size for the chosen number of candidate predictors [12]. Our models, particularly PREP-L and also PREP-S by 48 hours, validated well in external populations despite variations in country, setting and management. This suggests the models are transportable and potentially useful for other non-UK populations.

We only assessed predictors that are available in current everyday practice in high-resource settings, which may be seen as a weakness. Differences in management could affect the performance of the model. Although the models included predictors of composite outcome, we were unable to identify those associated with specific outcomes due to the small numbers of individual complications. The logistic model does not allow for predictions over time and therefore we also developed a survival model to provide risks at various time points including at 48 hours. However, we censored at 34 weeks, as one of the components of the outcome was delivery by 34 weeks. We were also unable to assess the added predictive contribution of biomarkers (sFlt1, sEng, PIGF) in maternal blood or urine, because the planned ASTRONAUT study which was going to provide data on biomarkers did not commence. We also could not differentiate iatrogenic premature births from spontaneous births. However, it is likely that pre-eclampsia and its complications, such as abruption, could predispose to spontaneous preterm labour [38]. Although admission to neonatal intensive care unit was considered an indication of morbidity by Delphi consensus [13, 39], its inclusion as a perinatal outcome may have weakened the prediction of other more clinically relevant outcomes. Due to the non-availability of all predictors in external datasets, we were unable to validate the original PREP models. However, our validation of reduced PREP-L and PREP-S models gives an insight into how the original models might perform. For example, a reduced PREP-L model showed good discrimination and calibration for predicting overall risk by discharge, and thus we expect the original PREP-L to have similar, if not better, performance when fully externally validated.

### Comparison to existing evidence

Current prediction models, such as PIERS and mini PIERS, included women with any onset pre-eclampsia, and not specifically those with early onset pre-eclampsia [4, 40]. Furthermore, existing models do not have sufficient sample size for predicting complications at 48 hours in women with early onset pre-eclampsia. They evaluated a large number of predictors for relatively fewer outcomes observed at 48 hours, and used the worst values of the tests, which may have overestimated the performance of the model [12]. Effective interventions such as delivery and anti-hypertensives could have contributed to the observed absence of traditional risk factors such as blood pressure, a crucial part of clinician’s risk assessment, in these models [4, 40].

The performance of the PIERS model was dominated by haematological complications, such as transfusion of any blood product or low platelet count, which contributed to about half of all complications. Transfusion of blood products scored the lowest for clinical importance in the management of women with early-onset pre-eclampsia, in a survey of experts [41]. Our PREP models’ predictions were heavily influenced by risk of early delivery before 34 weeks, a clinically more important outcome which helps inform mothers make informed decisions. Compared to the current PIERS model that had a C-statistic of between 0.7 and 0.8 at one week after admission, the PREP-L model showed discrimination of over 0.8 for a longer period until postnatal discharge from the hospital.

### Relevance to clinical care

Currently, mothers diagnosed with early onset pre-eclampsia in secondary care are transferred to tertiary hospitals. However, with only a third of these mothers suffering a complication by 48 hours, in current practice, many mothers diagnosed with the condition in secondary care would have been transferred to a tertiary centre. The promising discrimination and calibration performance of the PREP-S at 48 hours makes it suitable for use as a triage tool, in accurately identifying mothers for in utero transfer to tertiary care unit. It will also determine use of corticosteroids depending on the predicted probability of complications. With good agreement between the predicted and observed risk of complications in the PREP-S at 48 hours in the low- and intermediate-risk groups, women with a predicted probability of complications below 50% can avoid unnecessary transfer to tertiary units. Women categorised to be low risk by the PREP-L model could be followed-up in an outpatient setting, with high- and very high-risk women monitored as inpatients with regular intensive monitoring.

The model is available for everyday use in clinical practice as an excel sheet, and is being developed as an app to obtain individualised risk estimates for decisions on further management. Provision of personalised risk information allows parents to have the opportunity to discuss the expected outcomes. It is important to recognise that all prediction models in this field, including our PREP models, provides risk estimates in the context of current care and clinical management decisions. The models are not designed to guide clinicians’ decisions on choice of management such as timing of delivery, administration of anti-hypertensives and magnesium sulfate. A woman with a low predicted risk should be viewed as an individual with low outcome risk if current care pathways are used, as it may be the clinical care that results in her low-risk status.

### Relevance to research

The next stage in this prediction research is evaluation of the impact of using PREP models in clinical practice, in terms of whether they help improve subsequent maternal and perinatal outcomes; this requires well-planned robust randomised trials. The threshold for intervention, such as transfer to a tertiary unit or admission to the hospital and subsequent delivery, needs to be established by working with Patient and Public Involvement groups. The design for such clinical trials should be to randomise centres on use of PREP models as triaging tools to plan management in women with early onset pre-eclampsia. Evaluating the added value of biomarkers to our models may also be a subject of future research, though they have not been found useful in recent research [42]. This may be supported by individual patient data meta-analyses to improve power to identify important predictors.

## Conclusion

The PREP models enable the individualised risk prediction of complications in early-onset pre-eclampsia for overall risk and by 48 hours. They use routinely collected data and show promising performance upon internal and external validation. They can now be considered for use to support healthcare professionals and parents in making decisions on place of care, intensity of monitoring, and early in utero tertiary transfer to appropriate units.

## Additional file

Additional file 1: Table S1. Definition of the inclusion criteria for women recruited in the PREP study. **Table S2.** Crude univariable and multivariable analyses of candidate predictors and adverse fetal outcomes in women with early onset pre-eclampsia. **Table S3.** Definitions of the individual components of the composite outcomes evaluated in the PREP study*. **Table S4.** Changes since original application. **Table S5.** Rates of individual complications in women with early onset pre-eclampsia in the PREP study. **Table S6.** Characteristics of predictors available in the PREP study and external validation cohorts (PIERS and PETRA). (DOCX 43 kb)
