# Supplementary material for: Prediction of complications in early-onset pre-eclampsia (PREP): development and external multinational validation of prognostic models
Source: BMC Med. 2017 Mar 30;15:68. doi: 10.1186/s12916-017-0827-3 (PMC5372261; doi:10.1186/s12916-017-0827-3)
Supplement: Additional file 1: Table S1. — Definition of the inclusion criteria for women recruited in the PREP study. Table S2. Crude univariable and multivariable analyses of candidate predictors and adverse fetal outcomes in women with early onset pre-eclampsia. Table S3. Definitions of the individual components of the composite outcomes evaluated in the PREP study*. Table S4. Changes since original application. Table S5. Rates of individual complications in women with early onset pre-eclampsia in the PREP study. Table S6. Characteristics of predictors available in the PREP study and external validation cohorts (PIERS and PETRA). (DOCX 43 kb) [file 12916_2017_827_MOESM1_ESM.docx]

**ADDITIONAL FILE**

**Table S1: Definition of the inclusion criteria for women recruited in the PREP study**

| Inclusion criteria | Definition |
| --- | --- |
| New onset pre-eclampsia | New onset hypertension (systolic BP ≥ 140 mm Hg or diastolic BP ≥ 90 mm Hg on 2 occasions 4 -6 hours apart in women) after 20 weeks of pregnancy and new onset proteinuria (≥ 2+ in urine dipstick or PCR ratio of greater than 30mg/mmol or 300 mg of protein excretion in 24 hours).^51^ |
| Suspected pre-eclampsia | New onset hypertension (systolic BP ≥ 140 mm hg or diastolic BP ≥ 90 mm hg on 2 occasions 4 -6 hours apart in women) after 20 weeks of pregnancy, and 1+ proteinuria on urine dipstick |
| Superimposed pre-eclampsia |  |
| - In women with chronic hypertension and no proteinuria before 20 weeks’ gestation | New-onset proteinuria (as defined previously) |
| - In women with significant proteinuria before 20 weeks’ gestation   HELLP syndrome  One episode of eclamptic seizures without hypertension or proteinuria | Elevated serum alanine aminotransferase concentration (>70 U per litre) or worsening hypertension (either two diastolic BP of at least 110 mm Hg four hours apart or one diastolic measurement of at least 110 mm Hg if the woman had been treated with an antihypertensive drug), plus one of the following: increasing proteinuria, persistent severe headaches, or epigastric pain  Hemolysis, Elevated Liver enzymes, and Low Platelets syndrome: Presence of hemolysis based on examination of the peripheral smear, elevated indirect bilirubin levels, or low serum haptoglobin levels in association with significant elevation in liver enzymes and a platelet count below 100,000/mm(3) after ruling out other causes of hemolysis and thrombocytopenia.  Other neurological conditions of seizures have been excluded. |

Table S2: Crude univariable and multivariable analyses of candidate predictors and adverse fetal outcomes in women with early onset pre-eclampsia

| **Candidate predictors** | **No of women** ^[[1]](#footnote-1)^ | **No adverse fetal outcome**  **N=243** | **Adverse fetal outcome**  **N=702** | **Univariable model**  **N=945** | | | **Multivariable model**  **N=945** | | |
| --- | --- | --- | --- | --- | --- | --- | --- | --- | --- |
|  |  | **Mean (SD) or N (%)** | | **OR** | **95% CI** | **p** | **OR** | **95% CI** | **p** |
| **Maternal characteristics** | | | | | | | | | |
| Maternal Age in years | 943 | 31.0 (6.1) | 30.0 (6.1) | 0.972 | [0.949,0.995] | 0.018 | 0.984 | [0.954,1.015] | 0.301 |
| Log transformed gestational age at diagnosis | 945 | 3.4 (0.1) | 3.4 (0.1) | 0.889 | [0.446,0.978] | 0.007 | 0.911 | [0.393,0.987] | 0.014 |
| Multiple pregnancy (Reference: Singleton) | 945 | 225 (93%) | 637 (91%) |  |  |  |  |  |  |
| Twins |  | 17 (7%) | 62 (9%) | 1.288 | [0.738,2.250] | 0.373 | 1.676 | [0.862,3.260] | 0.128 |
| Triplets |  | 1 (0%) | 3 (0%) | 1.060 | [0.110,10.239] | 0.960 | 1.245 | [0.112,13.774] | 0.858 |
| Global test |  |  |  |  |  | 0.673 |  |  | 0.313 |
| **History** | | | | | | | | | |
| Score of medical history (Reference:0) | 944 | 116 (48%) | 478 (68%) |  |  |  |  |  |  |
| 1 medical history |  | 83 (34%) | 167 (24%) | 0.490 | [0.351,0.683] | <0.001 | 0.654 | [0.435,0.984] | 0.041 |
| 2 or more medical history |  | 44 (18%) | 56 (8%) | 0.309 | [0.198,0.481] | <0.001 | 0.434 | [0.246,0.767] | 0.004 |
| Global test |  |  |  |  |  | <0.001 |  |  | 0.009 |
| **Symptoms** | | | | | | | | | |
| Symptoms of Headache and/or visual disturbance | 919 | 102 (43%) | 278 (41%) | 0.891 | [0.658,1.206] | 0.456 | 0.812 | [0.552,1.193] | 0.289 |
| Symptoms of Epigastric pain, nausea and/or vomiting | 900 | 43 (19%) | 157 (23%) | 1.331 | [0.906,1.955] | 0.145 | 0.958 | [0.578,1.590] | 0.869 |
| Symptoms of Chest pain and/or dyspnoea | 821 | 12 (6%) | 48 (8%) | 1.340 | [0.668,2.687] | 0.410 | 1.190 | [0.502,2.822] | 0.693 |
| **Bedside examination and tests** | | | | | | | | | |
| Clonus | 545 | 10 (8%) | 84 (20%) | 2.399 | [1.262,4.562] | 0.008 | 1.487 | [0.609,3.633] | 0.384 |
| Exaggerated tendon reflexes | 594 | 20 (16%) | 126 (27%) | 2.063 | [1.318,3.231] | 0.002 | 0.852 | [0.461,1.575] | 0.610 |
| Systolic Blood Pressure | 941 | 153 (16) | 161 (20) | 1.025 | [1.016,1.034] | <0.001 | 1.005 | [0.992,1.019] | 0.414 |
| Diastolic Blood Pressure | 941 | 96 (11) | 101 (11) | 1.044 | [1.029,1.059] | <0.001 | 1.018 | [0.996,1.039] | 0.103 |
| Oxygen saturation: Abnormal (<94%) | 428 | 1 (1%) | 3 (1%) | 1.039 | [0.108,10.032] | 0.974 | 0.107 | [0.009,1.214] | 0.071 |
| Urine dipstick (Reference: None/trace) | 927 | 11 (5%) | 28 (4%) |  |  |  |  |  |  |
| 1+ |  | 71 (30%) | 98 (14%) | 0.549 | [0.256,1.179] | 0.124 | 0.609 | [0.259,1.435] | 0.257 |
| 2+ |  | 100 (42%) | 212 (31%) | 0.840 | [0.401,1.760] | 0.644 | 0.781 | [0.341,1.791] | 0.560 |
| 3+ |  | 42 (18%) | 261 (38%) | 2.429 | [1.117,5.284] | 0.025 | 1.445 | [0.587,3.555] | 0.424 |
| ≥4 |  | 13 (5%) | 91 (13%) | 2.739 | [1.111,6.751] | 0.029 | 0.974 | [0.334,2.840] | 0.961 |
| Global test |  |  |  |  |  | <0.001 |  |  | 0.045 |
| **Laboratory tests** | | | | | | | | | |
| Haemoglobin | 909 | 11.8 (1.1) | 12.0 (1.4) | 1.096 | [0.983,1.223] | 0.100 | 1.019 | [0.885,1.173] | 0.792 |
| Platelet Count | 905 | 244 (77) | 220 (77) | 0.996 | [0.994,0.998] | <0.001 | 0.998 | [0.996,1.001] | 0.128 |
| Log transformed ALT | 870 | 2.7 (0.6) | 3.0 (0.8) | 1.641 | [1.244,2.165] | <0.001 | 1.330 | [0.958,1.848] | 0.089 |
| Log transformed serum uric acid | 781 | 0.6 (1.4) | 1.0 (1.0) | 1.244 | [1.086,1.425] | 0.002 | 1.131 | [0.941,1.358] | 0.190 |
| Log transformed serum urea | 876 | 1.2 (0.4) | 1.4 (0.5) | 3.679 | [2.482,5.452] | <0.001 | 1.718 | [1.068,2.764] | 0.026 |
| Log transformed serum creatinine | 908 | 4.0 (0.3) | 4.1 (0.3) | 2.823 | [1.662,4.795] | <0.001 | 1.039 | [0.506,2.135] | 0.916 |
| Log transformed PCR | 837 | 3.9 (1.4) | 4.9 (1.4) | 1.569 | [1.397,1.762] | <0.001 | 1.290 | [1.111,1.497] | 0.001 |
| **Treatment provided** | | | | | | | | | |
| Antihypertensive therapy | 944 | 177 (73%) | 573 (82%) | 1.663 | [1.182,2.339] | 0.004 | 1.558 | [1.026,2.368] | 0.038 |
| MgSO4 administered | 944 | 9 (4%) | 135 (19%) | 6.190 | [3.100,12.363] | <0.001 | 2.402 | [1.036,5.573] | 0.041 |
| Steroids administered | 783 | 66 (41%) | 364 (59%) | 2.186 | [1.549,3.085] | <0.001 | 1.208 | [0.795,1.835] | 0.376 |
| **Ultrasound and cardiotocography** | | | | | | | | | |
| Uterine artery Doppler abnormal | 339 | 12 (14%) | 79 (31%) | 2.365 | [1.536,3.639] | <0.001 | 1.944 | [1.077,3.510] | 0.027 |
| CTG findings abnormal | 710 | 10 (6%) | 36 (7%) | 1.395 | [0.680,2.865] | 0.364 | 0.625 | [0.254,1.538] | 0.306 |
| Estimated fetal weight <10^th^ centile | 712 | 27 (15%) | 261 (49%) | 3.835 | [2.453,5.995] | <0.001 | 2.538 | [1.462,4.405] | 0.001 |
| Liquor volume abnormal | 890 | 10 (4%) | 46 (7%) | 1.548 | [0.776,3.087] | 0.215 | 1.279 | [0.519,3.152] | 0.593 |

*ALT: Alanine amino transferase; PCR: Protein Creatinine Ratio; MgSO4 Magnesium sulphate;*

*CTG: Cardiotocography*

**Table S3. Definitions of the individual components of the composite outcomes evaluated in the PREP study***

**a. Maternal**

| **Outcome** | **Definition** |
| --- | --- |
| Mortality | Maternal death at any time in pregnancy after delivery until discharge |
| Hepatic dysfunction | INR^[[2]](#footnote-2)^ >1.2 indicative of Disseminated Intravascular Coagulation (DIC) in the absence of treatment with Warfarin. (DIC is defined as having both: abnormal bleeding and consumptive coagulopathy (i.e., low platelets, abnormal peripheral blood film, or one or more of the following: increased INR, increased PTT^[[3]](#footnote-3)^, low fibrinogen, of increased fibrin degradation products that are outside normal non-pregnancy ranges)) |
| Hepatic hematoma or rupture | Blood collection under the hepatic capsule as confirmed by ultrasound or laparotomy |
| Glasgow coma score <13 | From GCS scoring system[^39^](#_ENREF_39) |
| Stroke | Acute neurological event with deficits lasting longer than 48 hours |
| Cortical Blindness | Loss of visual acuity in the presence of intact papillary response to light |
| Reversible Ischaemic Neurologic Deficit (RIND) | Cerebral ischaemia lasting longer than 24 hours but less than 48 hours revealed through clinical examination |
| Retinal detachment | Separation of the inner layers of the retina from the underlying retinal pigment epithelium (RPE, choroid) and is diagnosed by ophthalmological examination |
| Acute renal insufficiency | For women with an underlying history of renal disease: defined as creatinine >200 uM; for patients with no underlying renal disease: defined as creatinine >150 uM |
| Dialysis | Including haemodialysis and peritoneal dialysis |
| Transfusion of blood products | Includes transfusion of any units of blood products: fresh frozen plasma (FFP), platelets, red blood cells (RBCs), cryoprecipitate (cryo) or whole blood |
| Positive ionotropic support | The use of vasopressors to maintain a Systolic Blood Pressure > 90 mmHg or Mean Arterial pressure > 70 mmHg |
| Myocardial ischaemia/ infarction | Electrocardiography (ECG) changes (ST segment elevation or depression) without enzyme changes AND/OR any one of the following: 1) Development of new pathologic Q waves on serial ECGs. The patient may or may not remember previous symptoms. Biochemical markers of myocardial necrosis may have normalized, depending on the length of time that has passed since the infarct developed. 2) Pathological findings of an acute, healed or healing MI 3) Typical rise and gradual fall (troponin) or more rapid rise and fall (CK-MB) of biochemical markers of myocardial necrosis with at least one of the following: a) ischaemic symptoms; b) development of pathologic Q waves on the ECG; c) ECG changes indicative of ischaemia (ST segment elevation or depression); or d) coronary artery intervention (e.g., coronary angioplasty) |
| Require >50% oxygen for greater than one hour | Oxygen given at greater than 50% concentration based on local criteria for longer than 1 hour |
| Intubation other than for caesarean section | Intubation may be by ventilation, Electrical Impedance Tomography or Continuous Positive Airway Pressure |
| Pulmonary oedema | Clinical diagnosis with x-ray confirmation or requirement of diuretic treatment and SaO_2_ <94% |
| Postpartum Haemorrhage | >1L of blood loss after delivery |
| Early preterm delivery | Delivery at gestational age of less than 34 weeks |

b: Fetal and neonatal

| **Outcome** | **Definition** |
| --- | --- |
| Perinatal or infant mortality | Death of a fetus or neonate. Infant mortality is the death of a child less than one year of age |
| Bronchopulmonary dysplasia | Oxygen requirement at 36 weeks corrected gestation unrelated to an acute respiratory episode |
| Necrotising enterocolitis include only Bell's stage 2 or 3 | Evidence of pneumotosis intestinalis on abdominal x-ray and/or surgical intervention |
| Grade III/IV intraventricular haemorrhage | Bleeding into the brain's ventricular system, where ventricles are enlarged by the accumulated blood or bleeding extends into the brain tissue around the ventricles |
| Cystic periventricular leukomalacia | Softening and necrosis in the hemispheric white matter in newborns that may result from impaired perfusion at the interface between ventriculopetal and ventriculofugal arteries |
| Stage 3-5 retinopathy of prematurity | Abnormal blood vessel development in the retina of the eye, where blood vessel growth is severely abnormal, where there is a partially or totally retinal detached retina |
| Hypoxic ischaemic encephalopathy | Apgar score ≤ 5 at 10 mins and/or pH 7.00 in first 60 minutes of life and/or Base deficit ≥ -16 in first 60 minutes associated with abnormal conscious level (lethargy, stupor or coma) and seizures and/or poor/weak suck and/or hypotonia and/or abnormal reflexes. |

# Table S4: Changes since original application

| **What was proposed in original grant application** | **What was done in the PREP study** |
| --- | --- |
| 1. The original target sample size was 500 women with confirmed diagnosis of pre-eclampsia | The sample size was revised so we continued recruitment until 100 women had experienced an adverse events. The population did not change |
| 2. Update on maternal predictor variables | Chest pain and dyspnoea were added as candidate predictors. Gestational age, maternal age and platelet count were also added to the maternal prognostic factors |
| 3. One general list of candidate prognostic factors | Candidate prognostic factors were split into maternal and fetal predictor variables and only the fetal predictor variables included Ultrasound |
| 4. Symptoms of headache, epigastric pain, nausea, chest pain, dyspnoea or visual disturbance were one variable | These were split and regrouped into  1) symptoms of headache and visual disturbance  2) epigastric pain and nausea  3) chest pain and dyspnoea; forming 3 variables relating to a particular body system |
| 5. Blood pressure was one variable | This was split into systolic blood pressure and diastolic blood pressure |
| 6. Outcome assessment by 48 hours and by discharge | In the logistic model we had insufficient sample size to assess model performance at 48 hours. Hence we developed a second model, the survival model to provide risks at various time points including 48 hours. However, we censored at 34 weeks, as one of the components of the outcome is delivery by 34 weeks |
| 7. Develop the PREP model in the ASTRONAUT cohort of women | The ASTRONAUT study did not commence, and hence we were unable to work on its data |
| 8.Validate the PREP model in PIERS and PETRA cohort | We validated rPREP-L in both external datasets. We were unable to validate the rPREP-S model in the PETRA dataset, dates and times of outcome occurrence were not reported |
|  |  |
| 9. Assess the added predictive contribution of biomarkers (sFlt1, sEng, PIGF) in maternal blood or urine. | The ASTRONAUT study planned to provide data on biomarkers did not commence, and hence we were unable to work on its data |
| 10. Update of maternal outcomes | Platelet count and infusion of any third parenteral  antihypertensive removed as maternal outcomes. Preterm delivery <34 weeks added as a maternal outcome. |

Table S5: Rates of individual complications in women with early onset pre-eclampsia in the PREP study

**a. Maternal complications**

| **Adverse maternal outcome** | **No. of women with complications**  **N=946**  **n (%)** |
| --- | --- |
| Maternal death | - |
| **Neurological** |  |
| Eclamptic Seizures | 12 (1.3%) |
| Glasgow Coma score | 3 (0.3%) |
| Stroke or Reversible Ischaemic Neurological Deficit (RIND) | - |
| Cortical blindness | - |
| Retinal detachment | - |
| Posterior reversible encephalopathy | 2 (0.2%) |
| Bells palsy | - |
| **Hepatic** |  |
| Hepatic dysfunction | 12 (1.3%) |
| Subcapsular haematoma | - |
| Hepatic capsule rupture | - |
| **Cardiorespiratory** |  |
| Need for positive inotrope support | 1 (0.1%) |
| Myocardial ischaemia or infarction | - |
| At least 50% forced inspiratory oxygen (FIO_2_) for greater than 1 hr | 7 (0.7%) |
| Intubation | 9 (1.0%) |
| Pulmonary oedema | 6 (0.6%) |
| **Renal** |  |
| Acute renal insufficiency | 5 (0.5%) |
| Dialysis | 5 (0.5%) |
| **Haematological** |  |
| Blood transfusion | 51 (5.4%) |
| Abruptio placentae | 25 (2.6%) |
| Postpartum haemorrhage | 74 (7.8%) |
| **Preterm delivery** |  |
| Delivery at less than 34wks gestational age | 580 (61.3%) |
| *At least one of the above occurred by discharge* | *633 (66.9%)* |
| *At least occurred before 34 weeks of gestational age* | *584 (61.7%)* |

**b. Fetal and neonatal complications**

| **Adverse fetal outcome** | **No. of pregnancies with complications**  **N=945**  **n (%)** |
| --- | --- |
| Stillbirth | 16 (1.7%) |
| Neonatal death | 23 (2.4%) |
| Bronchopulmonary dysplasia | 41 (4.3%) |
| Necrotising enterocolitis | 34 (3.6%) |
| Grade III/IV intraventricular haemorrhage | 11 (1.2%) |
| Cystic periventricular leukomalacia | 5 (0.5%) |
| Stage 3-5 retinopathy | 7 (0.7%) |
| Hypoxic ischaemic encephalopathy | 2 (0.2%) |
| Admission to Neonatal Intensive Care Unit at any time | 681 (72.1%) |
| *At least one of the above occurred by discharge* | 702 (74.3%) |

Table S6: Characteristics of predictors available in the PREP study and external validation cohorts (PIERS and PETRA)

| **Characteristics of women** | | |  | | **PREP** |  | **PIERS** |  | **PETRA** |
| --- | --- | --- | --- | --- | --- | --- | --- | --- | --- |
|  |  |  | **Available data** | | **n=954** | **Available data** | **n=634** | **Available data** | **n=216** |
| Gestational age at diagnosis | Mean (SD) | | 954 | | 30.5 (2.9) | 634 | 30.2 (3.0) | 216 | 29.4 (2.6) |
| **Maternal characteristics** | | | | | | | | | |
| Maternal age | | Mean (SD) | | 952 | 30.2 (6.1 | 634 | 31.2 (6.3) | 216 | 30.0 (5.0) |
| Number of fetuses in pregnancy | | Singleton | | 954 | 866 (91%) | 634 | 542 (85%) | 216 | 216 (100%) |
|  |  | Twins | |  | 83 (9%) |  | 88 (14%) |  | - |
|  |  | Triplets | |  | 5 (1%) |  | 4 (1%) |  | - |
| **History** | | | | | | | | | |
| Summary score for medical history | | 0 | | 953 | 601 (63%) | 634 | 284 (45%) | 216 | 182 (84%) |
|  |  | 1 | |  | 251 (26%) |  | 251 (40%) |  | 30 (14%) |
|  |  | 2 or more | |  | 101 (11%) |  | 99 (15%) |  | 4 (2%) |
| **Symptoms** | | | | | | | | | |
| Symptoms of headache and/or visual disturbance | | Present | | 926 | 382 (41%) | 634 | 319 (50%) | - | - |
| Symptoms of epigastric pain, nausea and/or vomiting | | Present | | 907 | 202 (22%) | 634 | 220 (35%) | - | - |
| Symptoms of chest pain and/or dyspnoea | | Present | | 828 | 60 (7%) | 634 | 42 (7%) | - | - |
| **Examination** | | | | | | | | | |
| Clonus^[[4]](#footnote-4)^* | Present | | | 551 | 95 /17%) | - | - | - | - |
| Exaggerated tendon reflexes^[[5]](#footnote-5)^* | Present | | | 601 | 139 (15%) | - | - | - | - |
| Systolic blood Pressure | Mean (SD) | | | 949 | 159 (19) | 634 | 168 (20) | 216 | 157 (18) |
| Diastolic blood Pressure | Mean (SD) | | | 949 | 99 (12) | 634 | 105 (11) | 216 | 104 (11) |
| Oxygen saturation by Pulse Oximetry | Mean (SD) | | | 433 | 98.1 (1.6) | 474 | 96 (2) | - | - |
| Oxygen saturation: Abnormal (<94) | Present | | | 433 | 4 (1%) | 474 | 72 (15%) | - | - |
| **Laboratory tests** | | | | | | | | | |
| Haemoglobin | Mean (SD) | | | 917 | 11.9 (1.3) | - | - | - | - |
| Platelet Count | Mean (SD) | | | 913 | 226 (78) | 630 | 204 (77) | 215 | 172 (87) |
| Alanine amino transferase | Mean (SD) | | | 879 | 31.0 (71.0) | 630 | 65.5 (157.6) | 207 | 79.9 (139.3) |
| Aspartate amino transferase | Mean (SD) | | | 275 | 36.9 (61.1) | 600 | 74.3 (196.5) | 212 | 91.9 (160.7) |
| Serum uric acid | Mean (SD) | | | 789 | 0.6 (2.7) | - | - | - | - |
| Serum urea | Mean (SD) | | | 884 | 4.6 (4.4) | - | - | - | - |
| Serum creatinine | Mean (SD) | | | 916 | 61.9 (17.8) | 626 | 69.3 (20.5) | 214 | 67.8 (16.8) |
| Urine dipstick | None/Trace | | | 935 | 39 (4%) | 613 | 129 (21%) | - | - |
|  | 1+ | | |  | 170 (18%) |  | 69 (11%) | - | - |
|  | 2+ | | |  | 314 (34%) |  | 111 (18%) | - | - |
|  | 3+ | | |  | 306 (33%) |  | 141 (23%) | - | - |
|  | ≥4 | | |  | 106 (11%) |  | 163 (27%) | - | - |
| Urine PCR | Mean (SD) | | | 845 | 273 (492) | 437 | 276 (437) | - | - |
| **Treatment provided** | | | | | | | | | |
| Anti-hypertensive therapy at baseline | | Present | | 948 | 753 (79%) | 634 | 551 (87%) | 216 | 123 (57%) |
| Magnesium sulphate administration at baseline | | Present | | 948 | 144 (15%) | 634 | 325 (51%) | 216 | 34 (16%) |

1. *Descriptive of predictors based on the original non-imputed data. N is the number of women with available data* [↑](#footnote-ref-1)
2. International Normalised Ratio [↑](#footnote-ref-2)
3. Partial thromboplastin time

   * Adapted from the PIERS study [↑](#footnote-ref-3)
4. * Predictor is part of the survival model only and used as components of the final PREP model [↑](#footnote-ref-4)
5. [↑](#footnote-ref-5)
